# Ionizing Radiation Stimulates Expression of Pro-Osteoclastogenic Genes in Marrow and Skeletal Tissue

**DOI:** 10.1089/jir.2014.0152

**Published:** 2015-06-01

**Authors:** Joshua S. Alwood, Mohammad Shahnazari, Betsabel Chicana, A.S. Schreurs, Akhilesh Kumar, Alana Bartolini, Yasaman Shirazi-Fard, Ruth K. Globus

**Affiliations:** Bone and Signaling Laboratory, Space Biosciences Division, NASA Ames Research Center, Moffett Field, California.

## Abstract

Exposure to ionizing radiation can cause rapid mineral loss and increase bone-resorbing osteoclasts within metabolically active, cancellous bone tissue leading to structural deficits. To better understand mechanisms involved in rapid, radiation-induced bone loss, we determined the influence of total body irradiation on expression of select cytokines known both to stimulate osteoclastogenesis and contribute to inflammatory bone disease. Adult (16 week), male C57BL/6J mice were exposed to either 2 Gy gamma rays (^137^Cs, 0.8 Gy/min) or heavy ions (^56^Fe, 600MeV, 0.50–1.1 Gy/min); this dose corresponds to either a single fraction of radiotherapy (typical total dose is ≥10 Gy) or accumulates over long-duration interplanetary missions. Serum, marrow, and mineralized tissue were harvested 4 h—7 days later. Gamma irradiation caused a prompt (2.6-fold within 4 h) and persistent (peaking at 4.1-fold within 1 day) rise in the expression of the obligate osteoclastogenic cytokine, receptor activator of nuclear factor kappa-B ligand (*Rankl*), within marrow cells over controls. Similarly, *Rankl* expression peaked in marrow cells within 3 days of iron exposure (9.2-fold). Changes in *Rankl* expression induced by gamma irradiation preceded and overlapped with a rise in expression of other pro-osteoclastic cytokines in marrow (eg, monocyte chemotactic protein-1 increased by 11.9-fold, and tumor necrosis factor-alpha increased by 1.7-fold over controls). The ratio, *Rankl/Opg*, in marrow increased by 1.8-fold, a net pro-resorption balance. In the marrow, expression of the antioxidant transcription factor, *Nfe2l2*, strongly correlated with expression levels of *Nfatc1*, *Csf1*, *Tnf*, and *Rankl*. Radiation exposure increased a serum marker of bone resorption (tartrate-resistant acid phosphatase) and led to cancellous bone loss (16% decrement after 1 week). We conclude that total body irradiation (gamma or heavy-ion) caused temporal elevations in the concentrations of specific genes expressed within marrow and mineralized tissue related to bone resorption, including select cytokines that lead to osteoclastogenesis and elevated resorption; this is likely to account for rapid and progressive deterioration of cancellous microarchitecture following exposure to ionizing radiation.

## Introduction

During a spaceflight beyond the Earth's protective magnetosphere, astronauts are exposed to a complex mixture of ionizing radiation (Durante and Cucinotta 2011), including low-linear energy transfer (LET) gamma rays and protons, as well as more damaging high-LET radiation. Exposure to space radiation is characterized by relatively low doses (≤2 Gy) of ion species due to solar particle events (Shurshakov and others 1999; Parsons and Townsend 2000) or galactic cosmic rays (Zeitlin and others 2013; Hassler and others 2014). Simulated space radiation at these doses causes a rapid net decline in cancellous bone volume to total volume as well as a decrement relative to age-matched controls (Kondo and others 2009; Willey and others 2010). The bone loss is associated with increased osteoclast numbers and resorbing surfaces of osteoclasts lining trabeculae (Hamilton and others 2006; Alwood and others 2010; Kondo and others 2010; Willey and others 2010; Yumoto and others 2010; Lloyd and others 2012). Doses in the range of 1–2 Gy also are relevant to radiotherapy; total therapeutic doses can vary, but total body doses of 10–15 Gy typically are fractionated into single doses of ∼2 Gy, which ultimately can lead to increased fracture incidence (Baxter and others 2005). Exposure to radiation, particularly high-LET particles, has the potential to exacerbate the deleterious effects of musculoskeletal disuse, which occurs during prolonged bed rest or spaceflight (LeBlanc and others 2000; Lang and others 2004, 2006; Keyak and others 2009; Alwood and others 2010; Yumoto and others 2010).

Previous work shows that radiation exposure elicits a transient (<2 weeks) pro-resorptive state in cancellous tissue, resulting in net bone loss compared with basal (time of radiation) and age-matched controls (Kondo and others 2010; Willey and others 2010; Alwood and others 2012). Bone-resorbing osteoclasts are thought to cause the rapid (Kondo and others 2009; Willey and others 2010; Turner and others 2013) cancellous strut losses following simulated space irradiation, leading to loss of microarchitectural integrity (Alwood and others 2010). Radiation exposure transiently and markedly increases the numbers of osteoclasts and the extent of cancellous surfaces covered by osteoclasts. However, the role that receptor activator of nuclear factor kappa-B ligand (*Rankl*), the principal osteoclastogenic cytokine, plays in concert with other pro-osteoclastic inflammatory cytokines (Kim and others 2006; Takayanagi 2007; Boyce and Xing 2008) and oxidative stress is not fully understood with respect to the cancellous bone loss following radiation exposure (Kondo and others 2009).

Total body irradiation (≤2 Gy) causes time-dependent changes in the bone formation by osteoblasts, although these changes do not account for the early and rapid decrement in bone mass observed following exposure to total body irradiation within this dose range. Within 3 days of exposure to radiation, the net bone formation rate to bone surface (BFR/BS) is unaffected (Kondo and others 2010). Although radiation does affect osteoblast function within the first week of exposure, as indicated by an observed increase in mineral apposition rate (MAR), a concomitant reduction in the mineralizing surface yields no net change in the bone formation rate (Kondo and others 2010). Similarly, Willey and others (2010) also report increased MAR within the first week after exposure, but subsequently BFR/BS declines by 2 and 3 weeks after exposure to 2 Gy X-ray, while Turner and others reported transient increased bone formation even after very high doses of radiation (6 Gy). Therefore, radiation-induced changes in bone formation are more likely to contribute to later remodeling deficits rather than acute bone loss.

To better understand net bone loss in the acute response to radiation exposure, we examined the temporal expression within both marrow and mineralized tissue of *Rankl* and select pro-osteoclastogenic cytokines implicated in various models of inflammatory bone loss (Braun and Schett 2012) following exposure to low- or high-LET species of radiation. Furthermore, we investigated the temporal expression of a key antioxidant transcription factor (nuclear factor, erythroid-derived 2,-like 2, *Nfe2l2*), which is responsible for mounting a rapid defense against oxidative challenges (Kobayashi and Yamamoto 2005) and is known to play a role in dampening osteoclastogenesis (Hyeon and others 2013).

We hypothesized that radiation exposure induces expression of pro-osteoclastogenic, pro-inflammatory, and antioxidant genes within both marrow and mineralized tissue compartments and increases markers of bone resorption. Furthermore, we hypothesize that these changes are likely to contribute to later cancellous bone loss. This work shows an acute and time-dependent elevation of *Rankl*, monocyte chemotactic protein-1 (*Mcp1*), and tumor necrosis factor-alpha (*Tnf*) gene expression in the marrow or in skeletal compartments due to low- or high-LET irradiation. These molecular changes precede (<3 days) the manifestation of bone loss (3–7 days) following iron irradiation at a dose relevant to fractionated radiotherapy or space missions.

## Materials and Methods

### Animals

Post-pubescent (16 weeks±4 days at time of irradiation), male C57BL/6J mice (Jackson Laboratories) were individually housed and provided food (LabDiet 5001) and water *ad libitum*, as described elsewhere (Yumoto and others 2010). Animals were euthanized by CO_2_ inhalation or anesthetized with isoflurane, followed by blood draw through cardiac puncture. The Institutional Animal Care and Use Committees for NASA Ames Research Center and Brookhaven National Laboratory approved all procedures.

### Experiment design and radiation exposure

Experiments were conducted to determine the temporal changes in the levels of key genes and circulating proteins related to bone resorption in the latency period before the onset of overt structural loss. To evaluate heavy-ion effects, conscious mice were exposed to high-LET iron ions (^56^Fe, 600 MeV/ion, 5cGy or 2 Gy, at a rate of 5 or 0.50–1.10 Gy/min, respectively) at the NASA Space Radiation Laboratory, Brookhaven National Laboratory, or were sham irradiated as previously described (*n*=5–8/group). Mice were euthanized and tissues harvested at baseline (ie, time of irradiation) or 3 or 7 days after exposure. To evaluate gamma radiation effects, conscious mice were irradiated with low-LET ^137^Cs gamma rays [2 Gy, 0.80 Gy/min, as described in detail in Kondo and others (2010)] or were sham irradiated. Euthanasia and tissue harvest took place 4 h or 1 day (±2 h), 3 days, or 7 days after exposure (*n*=5–7/group).

### Bone microarchitecture

Bone volume and microarchitecture of the proximal tibial metaphysis were quantified by microcomputed tomography (6.8 μm pixel size, 3,500 ms integration time, 50 kV, Skyscan 1174; Bruker microCT) similar to Kondo and others (2009). Briefly, a 1.0-mm thick region located 0.24 mm distal to the proximal growth plate of the tibia was selected and semiautonomously contoured to include cancellous tissue. To assess bone loss, the bone volume to total volume fraction (BV/TV, %), trabecular thickness (Tb.Th, μm), trabecular number (Tb.N, 1/mm), and trabecular separation (Tb.Sp, μm) were calculated and reported following conventional guidelines (Bouxsein and others 2010).

### qRT-PCR for gene expression within marrow and skeletal tissue

Femora and tibiae were dissected, cleaned of soft tissues, flushed of bone marrow with phosphate-buffered saline, and stored in RNALater (Qiagen, Inc.) at −80°C. Bone marrow cells were lysed and preserved with guanidine-thiocyanate-containing RLT buffer (Qiagen, Inc.) with 1% beta-mercaptoethanol at −80°C. RNA was extracted from homogenized bone or marrow lysates using Trizol (Ambion), QIAshredder, and an RNeasy mini kit (Qiagen, Inc.). For each tissue, RNA was treated with an RNase-free DNase set (Qiagen, Inc.) in accordance with the manufacturer's instructions. RNA quality and quantity were determined using a spectrophotometer (NanoDrop). The RNA quality was confirmed by electrophoresis using the 2100 Bioanalyzer (Agilent Technologies).

Following the manufacturer's recommendations, RNA was reversed transcribed and simultaneously used for qPCR using the GoTaq^®^ Probe 1-Step RT-qPCR system (Promega). Portions of the following mouse gene sequences were amplified using TaqMan gene expression assays (Applied Biosystems, Inc.): receptor activator of nuclear factor kappa-B ligand (*Rankl*, assay ID: Mm00441906_m1), Osteoprotegerin (*Opg*, assay ID: Mm01205928_m1), Tumor necrosis factor-alpha (*Tnf*, assay ID: Mm00443260_g1), monocyte chemotactic protein-1 (*Mcp1*, assay ID: Mm00441242_m1), interleukin-6 (*Il6*, assay ID Mm00446190_m1), tartrate-resistant acid phosphatase (*Acp5*, assay ID: Mm00475698_m1), cathepsin-K (*Ctk*, assay ID: Mm00484039_m1), nuclear factor of activated T-cells, cytoplasmic 1 (*Nfatc1*, assay ID: Mm00479445_m1), nuclear factor, erythroid-derived 2,-like 2 (*Nfe2l2*, assay ID: Mm00477784_m1), and colony-stimulating factor 1 (*Csf1*, assay ID: Mm00432686_m1). We standardized gene expression levels to mitochondrial ribosomal protein L19 (*L19*, assay ID: Mm02601633_g1) to correct for differences in cellular input and RNA quality and facilitate comparison between samples. Hypoxanthine–guanine phosphoribosyltransferase (*Hprt1*, assay ID: Mm01545399_m1) and transmembrane protein 40 (*Tmem40*, assay ID: Mm00460636_m1) also were analyzed as alternate housekeeping genes. The reactions were performed in the 7300 RT-PCR system (Applied Biosystems) or SmartCycler real-time PCR system (Cepheid).

We analyzed multiple candidate housekeeping genes for normalization, including *L19*, *Hprt1*, and *Tmem40*. Gamma radiation exposure did not modulate levels of the gene *L19* at the various time points, but transiently and modestly increased gene expression of *Hprt1* (-0.4 cycles, 1.4-fold) and *Tmem40* (-1 cycle, 2.3-fold). Following iron irradiation, *L19* (as well as *Hprt1*) showed small increases in cycle number due to treatment (-0.4 cycles, 1.4-fold for *L19*). As these differences in housekeeping gene expression were small relative to those of cytokine and resorption marker levels, results reported were normalized relative to *L19* for both gamma and iron irradiation experiments.

### Serum tartrate-resistant acid phosphatase 5b

Blood was collected from the heart at the time of euthanasia and serum was separated and stored at −80°C until processed. Enzyme immunoassays were performed for measurement of tartrate-resistant acid phosphatase 5b (TRACP 5b), a biomarker for osteoclast-mediated bone resorption, using a commercial kit (Immunodiagnostic Systems) according to the manufacturer's protocol.

### Statistics

All data are reported as mean±standard deviation. To determine significant differences compared with sham-irradiated controls, a 1-way analysis of variance was used followed by Dunnett's *post hoc* test. If data were determined to be heteroscedastic (Bartlett's test), a Welch's ANOVA test followed by a *post hoc* test using the Bonferroni correction were used to determine significant differences from the sham-irradiated controls. Correlation analyses were performed on expression levels of select genes from marrow and bone specimens following gamma irradiation (for the sham and 1, 3, and 7 day endpoints) using the Pearson's product-moment coefficient (r). A *P* value ≤0.05 was accepted as significant for all analyses. All analyses were performed using JMP 8.0.2 (SAS).

## Results

### Cancellous microarchitecture following iron irradiation

**FIG. 1.**
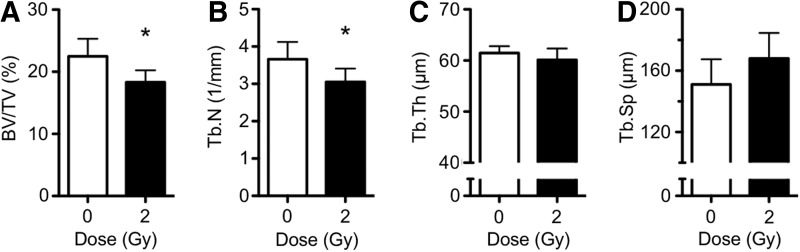
2 Gy iron irradiation caused acute bone loss in the tibial metaphysis by 7 days through removal of trabecular struts. **(A)** Bone volume fraction (BV/TV), **(B)** trabecular number (Tb.N), **(C)** trabecular thickness (Tb.Th), and **(D)** trabecular spacing (Tb.Sp). Data are mean±SD, with * denoting *P*<0.05 versus sham.

To determine the extent of bone loss over the short term, mice were irradiated with ^56^Fe ions (600 MeV) or were sham irradiated (0 Gy controls), then 7 days later bones were harvested and cancellous microarchitecture in the proximal tibia quantified *ex vivo* using 3D microcomputed tomography. Body weights of irradiated and control animals at the time of tissue harvest did not differ (data not shown). Compared with sham controls, irradiation with 2 Gy reduced BV/TV by 16%, Tb.N by 15%, and showed a trend of increased Tb.Sp by 11% (*P*=0.053, ANOVA), but did not affect trabecular thickness (Fig. 1). This is consistent with previous reports (Hamilton and others 2006; Alwood and others 2010). We included a basal group in our experiments showing BV/TV of 25.1%±3.6%, which was not different from the age-matched sham control group. A lower dose of iron (5 cGy) failed to elicit changes in bone structural parameters compared with controls and is therefore below the threshold dose for causing bone loss (data not shown). These results validate the model of ionizing radiation-induced cancellous bone loss.

### Marrow gene expression following iron irradiation

**FIG. 2.**
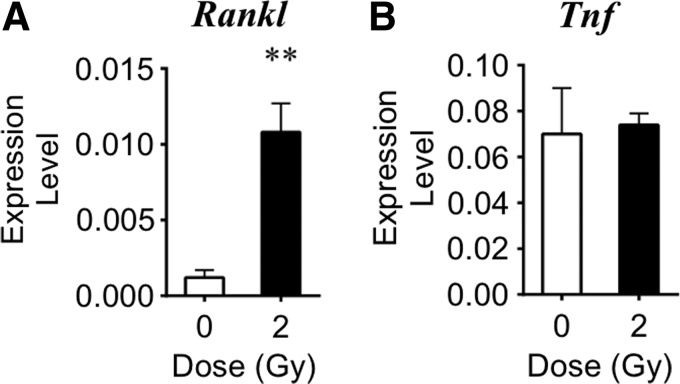
2 Gy iron irradiation effects on cytokine gene expression in tibial marrow cells on day 3 (expression level normalized to *L19*). Radiation exposure increased the gene expression levels of **(A)** *Rankl.* Gene expression levels of **(B)** *Tnf* were unchanged. Data are mean±SD, with ** denoting *P*<0.01 versus sham.

To determine if irradiation altered expression of various pro-osteoclastogenic genes, mRNA levels in bone marrow cell lysates were measured using quantitative RT-PCR 3 days after exposure to high-LET iron or sham irradiation. Within 3 days of exposure, iron irradiation increased expression of the *Rankl* gene by 9.2-fold compared with sham-irradiated controls (Fig. 2). At this time point, transcripts of *Opg* and *Mcp1* were not detected in the marrow (data not shown). Radiation exposure did not alter *Tnf* expression (Fig. 2). In contrast, a lower dose of iron (5 cGy) did not elicit changes in gene expression (data not shown). These data demonstrated that high-LET particulate irradiation with 2 Gy elicited pro-osteoclastogenic cytokine expression in the bone marrow tissue.

### Gene expression of skeletal tissue following iron irradiation

**FIG. 3.**
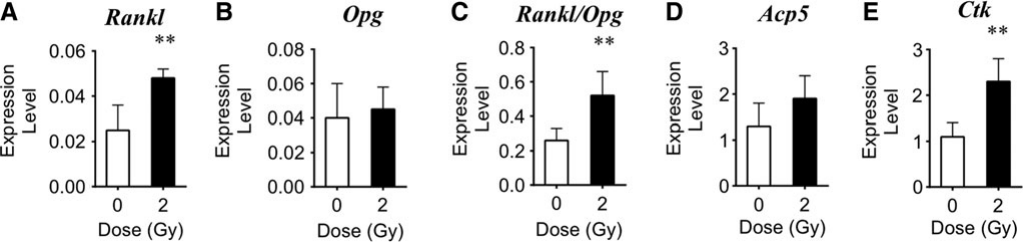
2 Gy iron irradiation effects on gene expression in tibial tissue (sans marrow) by day 3. Comparison of expression levels of **(A)** *Rankl*, **(B)** *Opg*, **(C)** *Rankl/Opg*, **(D)** *Acp5*, and **(E)** *Ctk* genes after iron irradiation compared with controls (expression level normalized to *L19*). Data are mean±SD, with ** denoting *P*<0.01 versus sham.

To determine if ionizing radiation altered expression of select genes related to osteoclastogenesis (*Rankl*, *Opg*) and osteoclast-mediated bone resorption (*Ctk*, *Acp5*) in cells within the mineralized compartment of skeletal tissue, RNA was purified from bone after removal of the marrow (leaving predominantly osteocytes). Within 3 days, iron irradiation increased expression of *Rankl* by 1.9-fold, *Acp5* by 1.5-fold, and *Ctk* by 2.1-fold over sham controls (Fig. 3). Expression levels of *Opg* did not change. The ratio of *Rankl/Opg* expression increased 2.8-fold, which provides a relative index that, on balance, cytokine levels favored increased bone resorption.

### Marrow gene expression following gamma irradiation

**FIG. 4.**
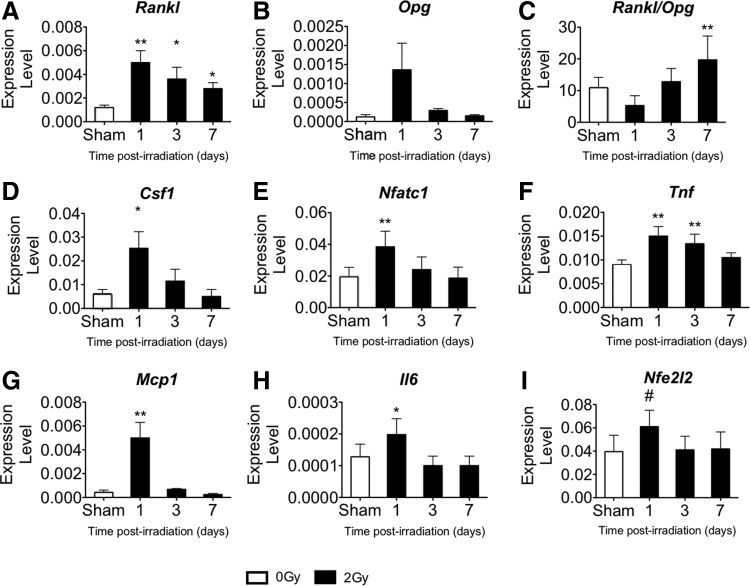
2 Gy gamma radiation increased expression of pro-osteoclastic and resorption-related genes in pooled tibial and femoral marrow (expression level normalized to *L19*). Time course (+1, +3, and+7 days post-irradiation) for the following genes compared with sham control: **(A)** *Rankl*, **(B)** *Opg*, **(C)** *Rankl/Opg*, **(D)** *Csf1*, **(E)** *Nfatc1*, **(F)** *Tnf*, **(G)** *Mcp1*, **(H)** *Il6*, and **(I)** *Nfe2l2*. Data are mean±SD, with * denoting *P*<0.05 and **P<0.01 versus sham and # denoting *P*=0.061 for the ANOVA.

To determine whether irradiation altered osteoclastogenic and inflammation-related genes, mRNA expression was measured in bone marrow at 4 h or 1, 3, or 7 days after low-LET gamma irradiation and in sham-irradiated controls. Within 4 h, 2 Gy gamma irradiation elevated *Rankl* in bone marrow by 2.6-fold over the sham; gene expression of *Opg* was undetectable regardless of treatment (data not shown). Subsequently, the expression of many genes of interest measured in bone marrow (*Rankl*, *Csf1*, *Nfatc1*, *Tnf*, *Mcp1*, and *Il6*) transiently increased within 1 day and subsequently declined toward sham levels (Fig. 4). Expression of *Tnf* remained elevated through day 3 post-irradiation, while high *Rankl* expression persisted through day 7 post-irradiation.

At their peak, expression of pro-osteoclastogenic genes, *Rankl* and *Csf1*, and the osteoclast-related transcription factor, *Nfatc1*, increased by 4.1-fold, 4.2-fold, and 2.0-fold, respectively (Fig. 4A, D, E); the *Rankl* decoy receptor *Opg* showed a trend toward increased expression by 11.3-fold (*P*=0.053 by ANOVA, Fig. 4B); pro-inflammatory genes *Tnf*, *Mcp1*, and *Il6* increased by 1.7-fold, 11.9-fold, and 1.6-fold, respectively (Fig. 4F, G, H), relative to controls. The ratio of *Rankl*/*Opg* increased by 1.8-fold at day 7 after irradiation (Fig. 4C). Additionally, radiation exposure showed a trend toward increased gene expression of *Nfe2l2* by 1.6-fold on day 1 compared with controls (*P*=0.061 for the ANOVA, Fig. 4I). Notably, levels of *Nfe2l2* expression were strongly and positively correlated with the osteoclast-related genes *Nfatc1* (*P*<0.05, *r*=0.89), *Csf1* (*P*<0.05, *r*=0.63), *Tnf* (*P*<0.05, *r*=0.62), and *Rankl* (*P*<0.05, *r*=0.56). These data show the temporal dependence of changes in cytokine gene expression in the marrow following radiation exposure.

### Gene expression of skeletal tissue following gamma irradiation

**FIG. 5.**
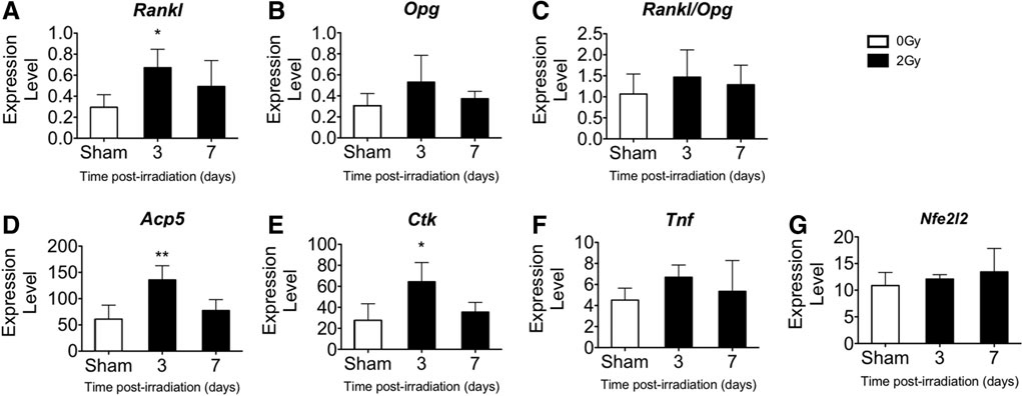
2 Gy gamma irradiation increased gene expression in femoral and tibial tissue (sans marrow) by day 3. Comparison of expression levels of **(A)** *Rankl*, **(B)** *Opg*, **(C)** *Rankl*/*Opg*, **(D)** *Acp5*, **(E)** *Ctk*, **(F)** *Tnf*, and **(G)** *Nfe2l2* genes after iron irradiation compared with controls (expression level normalized to *L19*). Data are mean±SD, with * denoting *P*<0.05 and ** denoting *P*<0.01 versus sham.

Following gamma irradiation, mRNA levels in femoral mineralized tissue (sans marrow) were quantified at 3 and 7 days after irradiation or sham using qRT-PCR normalized to the housekeeping gene, *L19*, as shown in Fig. 5. Within 3 days, gamma radiation exposure increased the expression of *Rankl* (2.3-fold), *Acp5* (2.2-fold), and *Ctk* (2.3-fold). Expression of *Tnf*, *Opg*, *Nfe2l2*, and the *Rankl/Opg* ratio was not changed (Fig. 5). These data suggest that bone-embedded and/or lining cells contribute to osteoclast stimulation and provide evidence for elevated expression of osteoclastogenic (*Rankl*) and resorption-related (*Acp5*, *Ctk*) genes in the skeletal tissue.

### Serum TRACP 5b following gamma irradiation

To determine if temporal changes in skeletal gene expression (Figs. 4 and 5) coincide with changes in a protein biomarker of resorption, the circulating levels of osteoclast-specific TRACP 5b were measured at 1, 3, and 7 days after gamma irradiation and in sham controls. Gamma irradiation increased TRACP 5b serum levels by 34% on day 1 and 17% on day 3 compared with sham, with a subsequent gradual decline toward control levels by day 7 (data not shown). Thus, circulating TRACP 5b levels showed a similar time course, although lower in magnitude compared with skeletal gene expression for *Rankl* and other pro-osteoclastogenic cytokines.

## Discussion

To better understand the mechanisms underlying radiation-induced stimulation of bone resorption, we investigated molecular signals within the latency period between radiation exposure and the manifestation of cancellous tissue loss. In summary, the results show that exposure to either gamma or heavy-ion radiation increased gene expression for the canonical osteoclastogenic factor, *Rankl*, as well as other pro-osteoclast cytokines (*Mcp1*, *Tnf*, *Il6*); this occurred in both marrow and mineralized tissue. These findings do not distinguish between changes in gene expression levels due to a shift in the cell population expressing osteoclastogenic cytokines, a generally higher level of expression for all the cells, or some combination of both. In fact, after exposure to radiation of sufficient dosage, there are pronounced, if transient, changes in marrow cell viability and increases in marrow macrophages, which are likely to contribute to the generation of new osteoclasts. Therefore, we interpret our results to show that exposure to ionizing radiation increased the tissue concentrations of mRNA levels generally so as to favor osteoclastogenesis.

Radiation-induced changes in cytokine expression were temporally related to changes in several indices of bone resorption, including gene expression (*Ctk*, *Acp5*, *Nfatc1*) and a serum biomarker (TRACP 5b levels). In addition, marrow expression of the global antioxidant transcription factor, *Nfe2l2*, showed high correlation with the expression levels of several osteoclast-related genes. These data indicate that the activation of oxidative defenses and of osteoclast formation was temporally associated in response to radiation exposure. Our previous work showed radiation-induced oxidative damage presents later, at 10 days after exposure (Kondo and others 2010). Taken together, our results indicate that *Nfe2l2*-related antioxidant defense pathways were insufficient to prevent oxidative damage in bone.

Changes in gene expression (as early as 4 h or 1 day) preceded bone loss, which manifested in these experiments by day 7 after exposure to 2 Gy iron, although in some cases such as gamma irradiation and lower doses of iron, decrements in cancellous bone volume can be observed as early as 3 days after exposure (Kondo and others 2009; Yumoto and others 2010). Furthermore, a dose threshold was observed, as 5 cGy iron exposure failed to elicit changes in cytokine gene expression in the marrow or cancellous bone structure (data not shown); these findings provide additional indirect evidence in support of the hypothesis that early induction of osteoclastogenic cytokine gene expression by biologically effective doses of radiation leads to cancellous bone loss.

Together, the results demonstrate that radiation-induced structural changes were associated with a marrow environment favoring stimulation of osteoclast differentiation and activity by *Rankl* and other inflammation-related cytokines as follows: Radiation increased gene expression levels for pro-osteoclastogenic signaling molecules (*Csf1*, *Rankl*, *Tnf*), as well as a trend toward increasing antiosteoclastogenic molecules (*Opg*), in the marrow and mineralized tissue of irradiated mice compared with sham controls. Iron irradiation elevated the ratio of *Rankl/Opg* in both the marrow and skeletal tissue by day 3, whereas after gamma irradiation, the ratio was elevated in the marrow at 4 h and then again at day 7.

A rapid stimulation of *Rankl* expression by irradiation in the marrow and bone compartment, potentially from hematopoietic lineage cells (Pacifici 2010, 2012; Fumoto and others 2014) or stromal lineage cells (Suda and others 1999; Boyle and others 2003), is consistent with studies that used radiotherapeutic doses and regimens. In young mice, a single dose of 5 or 10 Gy increased *Rankl/Opg* in whole femora within 3 days (Han and others 2014). Using mice deficient in the antioxidant transcription factor, *Nfe2l2*, radiation exposure at high dose (20 Gy) increased RNA expression for *Rankl* in cultured osteoblasts grown *ex vivo*, but this was not observed in cells from wild-type mice (Rana and others 2012). These findings suggest an *Nfe2l2*-mediated increase in expression of antioxidant enzymes may dampen bone resorption responses to radiation.

Furthermore, when a macrophage cell line (RAW264.7) capable of differentiating into osteoclast-like cells after RANKL treatment is exposed to ionizing radiation (2 Gy gamma), gene expression levels rise for β3 integrin, an adhesion receptor that is important for osteoclast differentiation, as well as receptor activator of nuclear factor kappa-B (*Rank*), the receptor for *Rankl* on osteoclasts (Yang and others 2012). In other *in vitro* work, irradiation with 2 or 4 Gy increases *Rankl* in differentiated, MC3T3-E1 osteoblast-like cells (Yang and others 2013). Our work is the first to demonstrate the time course of radiation-induced changes in expression of various osteoclastogenic cytokines following 2 Gy exposure (both low- and high-LET) with subsequent bone loss and within the upper range of space-relevant doses and types of radiation.

Ionizing radiation also increased tissue expression of pro-inflammatory, osteoclastogenic ligands, *Mcp1*, *Tnf*, and *Il6*, which are all factors generally thought to stimulate osteoclast activity (Takayanagi 2007) in the presence of RANKL (Kostenuik and Shalhoub 2001; Yu and others 2004; Kim and others 2005, 2006; Sul and others 2012; Liu and others 2013). In some reports, TNF may act independently of RANKL to stimulate osteoclastogenesis (Kobayashi and others 2000). Our results are consistent with other work showing that *in vivo* exposure to ionizing radiation leads to rapid, complex, and interrelated sequence of signals constituting an immune-related cytokine response in bone marrow (Schaue and McBride 2010; Willey and others 2011; Schaue and others 2012; Buchwald and Aurora 2013). At a very high dose (10 Gy), which is sufficient to ablate the bone marrow of hematopoietic cells, radiation causes bone loss related to elevated fractalkine expression by vascular endothelial cells, inflammatory cytokines *Tnf*, interleukin 1 beta, and interferon gamma, and recruitment of preosteoclasts (CD11b) (Han and others 2014). Additionally, the time course of cytokine and resorption-related gene expression shown in this study coincides with that of marrow cell death and repopulation (Otsuka and others 2008; Kondo and others 2010), suggesting a possible relationship between an expanded population of marrow macrophages clearing apoptotic cells and debris after irradiation, the differentiation of macrophages into osteoclasts, and increased resorption activity.

Taken together, these data lead us to propose a 2-stage process for radiation-induced osteoclastogenesis: first, radiation-induced gene expression of osteoclastogenic cytokines leads to enrichment of the marrow with osteoclast precursors (monocyte–macrophage, myeloid lineage cells) and drives differentiation into osteoclasts, and, second, that inflammation-related cytokines co-stimulate differentiating and mature osteoclasts.

As a functional measure of active osteoclasts, we provide evidence that 2 Gy gamma irradiation elevated circulating levels of osteoclast-specific TRACP 5b protein, indicative of increased bone resorption. In this work, the radiation-induced elevation in serum TRACP 5b returned to control levels by day 7 following exposure, These results are consistent with those reported by Willey and others (2010, 2011) showing that X-irradiation (2 Gy) of female mice increases circulating TRACP 5b levels 1, 3, and 7 days after irradiation.

Radiation-induced decrements in cancellous tissue observed in these experiments were consistent with our previous results. Acute cancellous bone loss temporally manifests on day 3 at a dose as low as 10 cGy iron (Yumoto and others 2010). Persistent structural decrements (lasting >1 week) manifest at doses above ∼50 cGy iron (Yumoto and others 2010) and 1 Gy gamma exposure (Hamilton and others 2006; Kondo and others 2010) before being overtaken, in the case of gamma irradiation, by age-related bone loss (Alwood and others 2012). Taken together, given the observed time course of skeletal gene expression and serum resorption markers, we conclude that the structural deficits arose from a spike in osteoclastogenic cytokine expression that follows exposure to ionizing radiation (Kondo and others 2009; Willey and others 2010).

Limitations of this work include the dose rate used to model space radiation. It is an open question whether lower dose rate exposures that constitute true space radiation stimulate osteoclasts and bone loss to the same extent as the exposures used here. In addition, other signaling molecules, including various other cytokines not studied here, are likely also to play a role in regulating bone resorption after challenge with ionizing radiation, while changes in osteoblast lineage cells are likely to be important for maintaining structural integrity at later times post-irradiation.

In conclusion, an improved understanding of the molecular response to radiation exposure may aid the development of biological treatments to mitigate potentially deleterious skeletal consequences during space flight.
